# Pre-gestational counselling for women living with CKD: starting from the bright side

**DOI:** 10.1093/ckj/sfae084

**Published:** 2024-03-22

**Authors:** Iara Da Silva, Alejandra Orozco-Guillén, Elisa Longhitano, José Aurelio Ballarin, Giorgina Barbara Piccoli

**Affiliations:** Nephrology Department, Germans Trias i Pujol University Hospital, Badalona, Barcelona, Spain; Department of intersive medical care, Isidro Espinosa de los Reyes National Perinatology Institute, Mexico City, Mexico; Nefrologia, University of Messina, Messina, Italy; Fundacion Puigvert, Barcelona, Spain; Centre Hospitalier Le Mans, Le Mans, France

**Keywords:** CKD progression, counselling, pregnancy in CKD, pregnancy planning, pre-term delivery

## Abstract

Pregnancy in women living with chronic kidney disease (CKD) was often discouraged due to the risk of adverse maternal–fetal outcomes and the progression of kidney disease. This negative attitude has changed in recent years, with greater emphasis on patient empowerment than on the imperative ‘non nocere’. Although risks persist, pregnancy outcomes even in advanced CKD have significantly improved, for both the mother and the newborn. Adequate counselling can help to minimize risks and support a more conscious and informed approach to those risks that are unavoidable. Pre-conception counselling enables a woman to plan the most appropriate moment for her to try to become pregnant. Counselling is context sensitive and needs to be discussed also within an ethical framework. Classically, counselling is more focused on risks than on the probability of a successful outcome. ‘Positive counselling’, highlighting also the chances of a favourable outcome, can help to strengthen the patient–physician relationship, which is a powerful means of optimizing adherence and compliance. Since, due to the heterogeneity of CKD, giving exact figures in single cases is difficult and may even be impossible, a scenario-based approach may help understanding and facing favourable outcomes and adverse events. Pregnancy outcomes modulate the future life of the mother and of her baby; hence the concept of ‘post partum’ counselling is also introduced, discussing how pregnancy results may modulate the long-term prognosis of the mother and the child and the future pregnancies.

## INTRODUCTION

Chronic kidney disease (CKD) is estimated to affect up to 3%–6% of women of childbearing age [1]. This prevalence may rise in the future due to increased maternal age, advances in fertility treatment, and the increase in obesity, diabetes and hypertension observed even in young people. Given the central role of the kidney in pregnancy, nephrologists should be aware of the success rate and the main potential problems in pregnancy in CKD. Counselling, at least on the fundamental issues regarding pregnancy in CKD, is increasingly needed, as is identifying the situations that may merit specialized further advice.

Reproductive health is a recognized human right. According to the World Health Organization (WHO), ‘human rights documents resting on the recognition of the basic rights of all women to decide freely and responsibly the number, spacing, and timing of their children and to have the information and means to do so, and the right to attain the highest standard of sexual and reproductive health’ [2].

This right is not limited to healthy women, but is shared by women with chronic diseases, including CKD; however, in the past, women in high-risk groups were often advised against pregnancy and this still occurs without women having been given clear information on the risks involved in pregnancy and the possibility of success [3].

It is generally agreed that pre-conception counselling should enable a woman to make an informed choice on whether or not to undertake a pregnancy and should prepare her, both physically and psychologically, if the option of undertaking a pregnancy is chosen. Nonetheless, no optimal approach for counselling has been established, and a single, structured, approach is probably not the right answer, considering that both pregnancy and chronic diseases are perceived differently by different cultures and by individuals (Table 1) [4].

**Table 1:** Pre-conception counselling for pregnancy in CKD according to European guidelines’ best practices.

Guidelines	For whom?	Is the content of pre-conception counselling described?	Is the pre-conception counselling team described?	Further suggestions
Italian best practices (2016–2023) [7, 12–16]	All women with CKD	Not described	Multidisciplinary team that includes a nephrologist and obstetrician	Pregnant women with SLE and immunologic diseases, dialysis and KT should be followed by a multidisciplinary team, if possible, in a third-level hospital. Women who have experienced a PE episode should be advised on the risks of recurrence and undergo a nephrology work-up
British guidelines (2019) [9]	All women with CKD	Advise on increased risk of complications. Genetic counselling when appropriate. Optimization of pharmacologic treatments, control of disease activity, blood pressure and glycaemia; pre-dialysis education in advanced CKD	Consultant obstetrician and nephrologist or physician with experience treating CKD	The provision of counselling depends upon local availability of expertise. Expert, multidisciplinary pre-pregnancy counselling for women with an eGFR <60 mL/min/1.73 m^2^, CKD progression, uncontrolled hypertension, nephrotic-range proteinuria, SLE nephritis, KT and previous adverse obstetric outcomes
Dutch guidelines (2022) [10]	All women with CKD	Advice on effect of pregnancy on the underlying kidney disease, timing of pregnancy, impact of CKD on pregnancy specifying, besides the ‘classic’ risks, the effects on women's life expectancy and quality of life	Nephrologist and materno-fetal specialist. Physicians with an ‘affinity’ for pregnancy in CKD	University hospital counselling and follow-up in advanced CKD, KT or autoimmune diseases. Counselling and follow-up in general hospitals for other cases
German guidelines (2022) [8]	All women with CKD	Warning against complications and advising on ways to optimize pre-conception maternal health	Interdisciplinary team in which gynaecologists collaborate with nephrologists	Warning against an increased risk of malformations, however not fully supported by the literature

SLE: systemic lupus erythematous, KT: kidney transplantation; PE: preeclampsia; eGFR: estimated glomerular filtration rate.

One of the problems encountered in the field is the imprecision of risk evaluation, due both to the heterogeneity of kidney diseases in pregnancy and to the differences in obstetric policies, potentially affecting some outcomes, such as late preterm delivery or caesarean section. Furthermore, baseline data, i.e. before the start of pregnancy, are not always available, thus making predictions difficult; predictive models, adapted to different contexts, would be of great utility.

The field of obstetric nephrology is relatively new, and uncertainty about what the outcomes of CKD in pregnancy are and which actions can be taken to minimize the risks in pregnancy make counselling both vitally important and extremely difficult [5–11].

In this review, we will limit our discussion to pre-conception counselling, aware that the most complex clinical and ethical choices are those faced by patients (and physicians) when an unplanned (and/or unwanted) pregnancy occurs or when an unexpected diagnosis of CKD is made during pregnancy. While some of the issues discussed in this review also apply to these extremely challenging situations, the emotional implications are often even deeper and should be faced with individualized clinical and, if requested, psychological care.

## COUNSELLING FOR PREGNANCY IN WOMEN LIVING WITH CKD: COMPARISON OF EUROPEAN GUIDELINES/BEST PRACTICES

At the time of the present review, four European societies have produced best practices or guidelines on pregnancy in patients with CKD [7–10, 12–16]. All discuss pre-conception counselling, and do so with different nuances, due to differences in cultural backgrounds and also linked to the evolution of knowledge on pregnancy in Table 1 CKD.

In chronological order, the Italian best practices on pregnancy in CKD, published in 2016, suggest that all women with CKD, even with normal kidney function and without comorbidity, hypertension or proteinuria, should be advised that their pregnancy is considered to be at higher risk, and followed up accordingly [7]. Counselling is not explicitly described in the context of a highly individualized approach [7]. The best practice on CKD is part of a series of several best practices, each focused on a specific phase or problem, which include dialysis, kidney transplantation, contraception and assisted fertilization, and follow-up after preeclampsia [7, 12–16].

The British guidelines, published in 2019, suggest that women with CKD considering pregnancy be offered pre-pregnancy counselling by a multidisciplinary team, including a consultant obstetrician and nephrologist or specialized physician, and recommend advice be given on the increased risk of complications, including preeclampsia, preterm birth, fetal growth restriction, admission to a neonatal unit and caesarean delivery [9]. Further guidance regarding disease stabilization (immunologic diseases and diabetes), blood pressure control, drug management and planning in anticipation of the approach to disease flares and hyperemesis, as well as education on dialysis in advanced CKD stages, is provided [9].

The Dutch guidelines, published in 2022, are similar, describing in detail a suggested counselling policy, contextualizing it to type of facility, including expert nephrologists and materno-fetal specialists in a university hospital for counselling in severe CKD, kidney transplantation or autoimmune diseases [10]. Conversely, they advise that patients with initial CKD without the risk conditions mentioned above should be followed up in general hospitals by physicians with an ‘affinity’ for pregnancy in CKD [10]. The guidelines further detail the content of counselling: the effect pregnancy may have on an underlying kidney disease, the timing of pregnancy and its impact on CKD, specifying, besides the ‘classic’ risks (preeclampsia, preterm delivery, etc.), the potential effect on women's life expectancy and quality of life [10].

The German guidelines, also published in 2022, discuss counselling, warning against complications (increased risk of complications in pregnancy such as miscarriage, preterm birth, preeclampsia, fetal growth restriction, preterm placental abruption and intrauterine fetal death, as well as the deterioration of renal function) and explaining how to optimize pre-conception maternal health [8]. Of note, they are the only ones citing the absence of ‘threshold values for kidney parameters beyond which women would be advised not to become pregnant’ [8]. In addition, the German guidelines are the only ones that cite an increased risk of malformations, which is however not shared by the other guidelines, and is only partially supported by the current literature on pregnancy in CKD patients, since malformations appear to be limited to hereditary kidney diseases, use of teratogenic drugs (not specific for CKD) or associated with diabetes, once more, not specifically linked to CKD [8].

## STARTING COUNSELLING FROM THE ‘BRIGHT SIDE’

All the guidelines and best practices mentioned above, even when specifying that pregnancy is now possible in all CKD stages and grades, face counselling either by identifying cases that should be followed up with particular care (Italian best practices and Dutch guidelines) or by listing the risks [7–10]. The German guidelines are the only ones that explicitly mention the possibility of advising against pregnancy, while stating that no specific threshold exists [8]. None of the guidelines start ‘on the bright side’, telling women with CKD who have sought counselling about the chances of conceiving, continuing pregnancy and delivering a healthy baby.

Hope is, however, part of care and a positive approach can help to establish a sound patient–physician relationship, which is an important determinant of adherence to prescriptions, and that is needed when undertaking the ‘eventful journey’ of pregnancy, not only in uraemic patients, but more widely in CKD [17–19].

Giving hope is, of course, not synonymous with giving simplistic over-expectations, and is overall associated with better health-related outcomes in several fields, although this has not yet been studied in CKD pregnancies [20–24].

**Figure 1:**
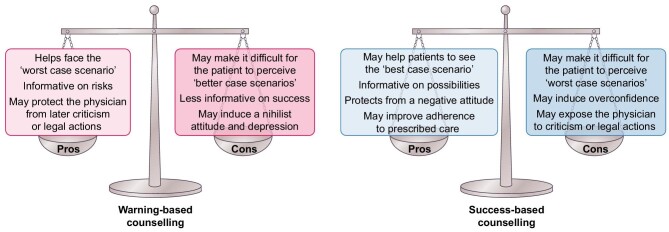
Pros and cons of warning-based counselling and success-based counselling.

The focus on risks partially derives from a defensive approach to counselling. The widespread idea is that the physician who underlines all risks is ‘protected’ from criticism, since the patient was ‘warned’ against the risks, and accepted them. However, the potential damage induced by a negative attitude, discouraging and frightening the patient who needs to decide what course to follow in a situation that is never entirely risk-free (even in healthy women), is not considered, either from a clinical point of view or from a legal one. Figure 1 summarizes some of these points. In the following paragraphs we will focus on fertility, barriers to conceiving, chances of success of pregnancy and pregnancy outcomes, trying to integrate classic ‘risk-based’ counselling with a balanced ‘positive’ approach (Fig. 1).

## FERTILITY AND BARRIERS TO PREGNANCY IN WOMEN LIVING WITH CKD

Counselling should make it possible to plan the best time for a woman to become pregnant and establish the rules for close monitoring before and during pregnancy. This can improve short- and long-term outcomes, as suggested by the few studies on this issue. For example, in a retrospective analysis of 179 women with CKD who received preconception counselling at a single centre, undertaken by a multidisciplinary team including an obstetrician and a nephrologist, about 90% of the respondents found the experience informative and helpful during pregnancy [26].

Conceiving is possible in all CKD stages, and in all chronic kidney diseases. However, fertility is reduced in advanced CKD, due to a combination of hormonal derangements, pharmacologic interference and psychological problems, often worsened by prejudices and social misconceptions. No threshold level of kidney function below which fertility starts declining has been set, and, as will be further discussed, recent data suggest that the probability of conceiving has been underestimated even in women on dialysis [25]. Conversely, kidney transplantation (KT) only partially restores fertility, and, even if the outcomes in women with good kidney function, and without immunological or clinical complications, are probably better than in advanced CKD and consistently better than on dialysis, this should also be considered in counselling, when examining the ‘ideal timing’ of pregnancy, as will be discussed below [1, 13].

If counselling starts on ‘the bright side’, the probability of getting pregnant and barriers to conception should be considered in the first place.

**Figure 2:**
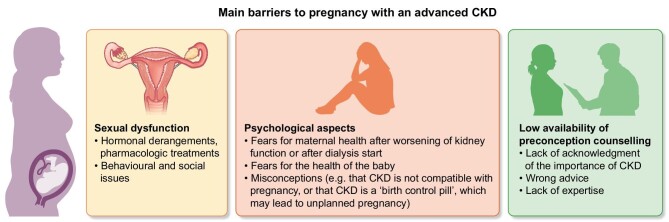
Main barriers to pregnancy in women living with an advanced kidney disease.

Figure 2 summarizes the main barriers faced by a woman with an advanced kidney disease who wants to become pregnant.

## SEXUAL DYFUNCTION IN WOMEN LIVING WITH CKD

**Figure 3:**
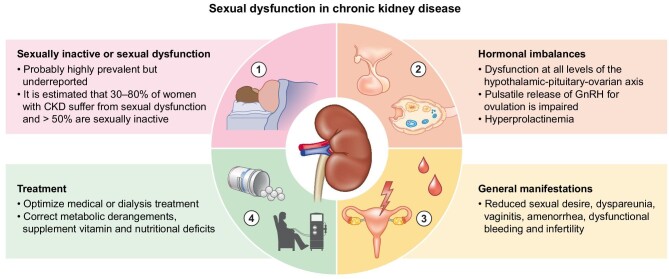
Sexual dysfunction in women with kidney disease.

Sexual dysfunction in women with CKD is probably common and under-recognized; it involves hormonal imbalances as well as psychological issues [27–29]. Women with advanced CKD or kidney failure may have alterations in the hypothalamic–gonadal axis, causing menstrual irregularities, abnormal uterine bleeding, anovulation and early menopause (Fig. 3) [27–29]. Conversely, in the absence of specific treatments that may reduce fertility, the paradigm of which is cyclophosphamide for lupus nephropathy, fertility is probably not reduced in early CKD [27–30]. There is little literature concerning fertility in early CKD, and it is unlikely that our knowledge will improve in the short term, since most early CKD cases are asymptomatic and are generally found only if specifically sought. Proof of this concept is the fact that CKD is discovered in up to 20% of pregnancies complicated by preeclampsia when a nephrology workup is performed [31, 32].

Becoming a mother is often challenging for women with advanced CKD since as kidney impairment progresses, fertility declines, and, probably, impairment of sexual life becomes more frequent while the risk of adverse fetal and maternal outcomes increases [33].

## CLINICAL AND PSYCHOLOGICAL BARRIERS IN WOMEN LIVING WITH CKD WHO WANT TO BECOME PREGNANT

The drive for procreation is a biological need in mammals. Legitimating this desire could be a first step in counselling: it can lead to discussion of anxiety about the future, which is dominant in some cases, while the desire to have a child as a substantial reinvestment against the disease is the driving force in others.

There are few studies that analyse CKD patients’ views on pregnancy; in two of these studies, both from Anglo-Saxon settings, decision-making was depicted as very demanding; one respondent described it as ‘emotional wrestling, influenced by the need to weigh a variety of risks that carried significant consequences for their health, family, job and lifestyle’ [34, 35]. Psychological barriers, including concerns that pregnancy will surely affect maternal kidney health or induce fetal malformations, play an important role in some cases, while in others a sense of responsibility towards a grafted organ is felt to be a major barrier against exposure to risk [34, 36, 37].

The balance between reassuring and describing risks, and between fear and confidence is complex. Some women affected by CKD are not even aware that they can become pregnant and give birth. Besides the content of the discussion, the way information is communicated is important, and should be adapted to the context and to the individual patient [35].

In some settings, a psychologist can offer support, clarify information and answer questions. However, the involvement of a psychologist may not always be welcome, and the principal ‘conductor’ of the consultation is often a nephrologist and/or obstetrician [37–39].

## LOW AVAILABILITY OF PRECONCEPTION COUNSELLING

While patients coming for counselling are already aware that they have CKD, and are considering pregnancy, many, if not most women with CKD do not undergo counselling. This occurs for a number of reasons.

First, many patients are unaware they have CKD. Since serum creatinine is not included in health screening before and during pregnancy, CKD is often diagnosed only when a woman develops complications in pregnancy [31, 32]. Hence, some experts hold that serum creatinine should be included in the basic pregnancy tests [40].

While pregnancy is a valuable occasion to diagnose CKD, in many parts of the world it is the only time a woman sees a doctor, and this usually happens during and not before pregnancy. In such cases, the problems of counselling merge with those of communicating the diagnosis of a (unexpected) disease [41].

Likewise, even in high-income countries (HIC), belonging to poorer strata of the population is associated with a higher probability of unplanned pregnancy, teenage pregnancy and adverse pregnancy outcomes. No data specific to the CKD population are presently available.

Second, there is a lack of expertise on the risks and on the management of pregnancy in women living with CKD; this lack of expertise is shared in HIC and low- and middle-income countries (LMIC), and is indeed understandable, when considering that the field of ‘obstetric nephrology’ is relatively new, and that while this subspecialty is widely developed in nephrology, it has no equivalent in obstetrics. Teaching programs, which are increasingly organized at the national or international level (including a training the trainees program of the International Society of Nephrology) will hopefully help to fill this gap.

## CHANCES OF SUCCESS AND MAIN RISKS OF ADVERSE PREGNANCY-RELATED EVENTS IN WOMEN LIVING WITH CKD

While a detailed review of the outcomes of pregnancy is beyond the scope of this review, we suggest that, since most of the available studies on patients’ views on pregnancy report messages of frailty and fear [34–38], a good starting point would be to explain that having a child is possible in any CKD stage, before starting to give a detailed list of the risks involved.

Table 2 reports some suggestions that can serve as the basis of a discussion with a patient, bearing in mind that what is probably most needed is a personalized clinical and psychological approach.

**Table 2:** Some questions that may need to be addressed in counselling for women living with CKD who consider pregnancy.

Item discussed	Available information	References
Probability of a live-born baby	Few data are available on early miscarriages (<20 gestational weeks); at least after KT they do not seem more frequent than in the overall population, while they may be more frequent in advanced CKD and dialysis. Intrauterine death is exceptional in patients on follow-up (same rates as in the overall populations in the few series reporting on this outcome)	[33, 42, 43]
Probability of a full-term baby (≥37 gestational weeks)	Possible in all CKD stages, including dialysis; however, probability decreases along with CKD stage	[33, 44, 45]
Probability of having a child without developmental anomalies	Possible in all CKD stages, including dialysis; the main risks are those related to prematurity, hence the need to plan for follow-up in settings where neonatal care facilities are available, in particular in high-risk cases	[33, 44, 45]
Probability of a baby without congenital anomalies	No indication of a higher incidence of malformations due to CKD, apart from those linked to hereditary kidney diseases and to teratogenic drugs (issues to be discussed at counselling). Diabetes is also associated with an increased risk of malformations, in relation to diabetes control and not to kidney function	[7, 8, 46, 47]
Probability of preserving kidney function	Kidney function is almost always preserved in patients with normal kidney function and without proteinuria or hypertension at baseline; decreased kidney function occurs more frequently in advanced CKD	[7, 48–51]

KT: kidney transplantation.

General and specific risks are discussed in Table 3. Once more, the risks reported in the table below can be ‘positively’ re-framed as barriers to success, and guides for intervention, bearing in mind however that while in experienced hands and adequate settings, risks can be reduced, offsetting the incidence of adverse pregnancy outcomes is still an unmet goal.

**Table 3:** Main adverse outcomes in pregnancies in women living with CKD (modified from [62]).

Term	Usual definition	Main issues
**Mother**		
Maternal death	Death in pregnancy or within 1 week to 1 month postpartum	Precise quantification remains elusive, particularly in HIC where the reported cases are mainly, if not exclusively, affected by active and uncontrolled immunologic disease, including SLE. In LMIC quantification is difficult also because of lack of diagnosis of CKD. The link with AKI is strict, and suggests an important role for (undiagnosed) CKD
CKD progression	Decrease in GFR, rise in sCr, shift to a higher CKD stage	Evaluated and estimated differently, it may be associated with obstetric policies, considering or not early delivery in the presence of deteriorating kidney function; particularly prevalent in advanced CKD, where it is reported in 20%–80%. There appears to be no notable rise in the earlier stages of CKD, but progression might be faster after a complicated pregnancy
PE and hypertensive disorders of pregnancy	Development of hypertension, accompanied by proteinuria or other signs of end-organ impairment	The definition of PE superimposed on CKD lacks consensus. In the absence of placental abnormalities, women with CKD might still experience hypertension and/or proteinuria. The diagnostic challenge could benefit from including placental biomarkers related to the angiogenesis–anti-angiogenesis axis
AKI	Usually employing the same criteria outside the context of pregnancy	AKI is more frequent if background CKD is present. Conditions like PE, HELLP syndrome, sepsis, and intrapartum haemorrhage can trigger AKI in the presence of CKD. Given the reduction in creatinine levels during pregnancy, having baseline kidney function data is imperative for early diagnosis
Immunologic flares and neonatal SLE	Flares of immunologic diseases in pregnancy	Previously believed to occur more frequently during pregnancy, especially in SLE, these risks are likely to apply primarily to individuals initiating pregnancy with an active disease or experiencing a recent flare-up. There is no unanimous consensus on defining a ‘safe’ zone; however, in cases of quiescent, well-controlled diseases, these risks do not seem to be more prevalent than in carefully disease-matched non-pregnant individuals
Transplant rejection	Acute rejection in pregnancy	Comparable to SLE, rejection episodes are not more prevalent than in carefully matched controls. They may occur more frequently in unplanned pregnancies, particularly in unstable patients
**Offspring**		
Abortion	Fetal loss, before 21–24 gestational weeks	The occurrence may be higher in advanced CKD, but data are lacking. It is a concern in immunologic diseases, potentially but not exclusively associated with the presence of LLAC, as well as in cases of diabetic nephropathy
Stillbirth	Delivery of a non-viable infant, after 21–24 gestational weeks	The risk is probably not increased in early CKD, but it may be a concern in advanced CKD and in dialysis patients. While not associated with extreme prematurity, it is increased in SLE, immunologic diseases, or diabetes, especially if the disease is not well controlled
Perinatal death	Death within 1 week to 1 month after delivery	Typically arising from extreme prematurity, it entails the risks of respiratory distress, neonatal sepsis and cerebral haemorrhage
Small, very small baby	A baby weighing <2500–1500 g at birth	The weight should also be calculated in relation to gestational age; risks may be lower for children adequate for gestational age
Preterm, early/extremely preterm	Delivery before 37–34 or 32–28 completed gestational weeks	The risk of preterm and early preterm delivery rises across stages of CKD. Extremely preterm births could become a significant concern in cases of undiagnosed or late-referred CKD and pregnancy-AKI
SGA and IUGR	<5th or <10th centile for gestational age	Closely and inversely associated with preterm delivery; SGA and IUGR are likely to correlate with the risk of hypertension, metabolic syndrome, and CKD in adulthood more than absolute birth weight
Malformations	Any kind of malformation	Congenital malformations do not appear to be more prevalent in CKD patients who are not treated with teratogen drugs. There is an exception in the case of diabetic nephropathy, where the increase is usually attributed to diabetes itself. Hereditary diseases, such as autosomal dominant ADPKD, reflux nephropathy and CAKUT may manifest at birth
Hereditary kidney diseases	Any kind of CKD	Various forms of CKD exhibit a hereditary pattern or predisposition. Alport's disease is one of the most common ones, but kidney tubular disorders, and mitochondrial diseases have a genetic basis; IgA nephropathy shows familial clustering in about 30% of cases
CKD—hypertension in adulthood	Higher risk of hypertension and CKD in adulthood, and globally of metabolic syndrome	The delayed maturation of nephrons leads to a reduced nephron count in preterm infants. The associated risks are generally higher in SGA and IUGR babies compared with preterm infants who are appropriate for their gestational age. The risks are also higher for all the components of the metabolic syndrome and for cardiovascular diseases. The risks are shared by all premature babies and are not CKD specific
Other long-term problems	Developmental disorders	Primarily linked to prematurity, cerebral haemorrhage or neonatal sepsis, they are a threat for all preterm infants, and are not CKD specific

SLE: systemic lupus erythematous; PE: preeclampsia; AKI: acute kidney injury; sCr: serum creatinine; HELLP: haemolysis, elevated liver enzymes, low platelet syndrome; SGA: small for gestational age; IUGR: intrauterine growth restriction; ADPKD: autosomal dominant polycystic kidney disease; CAKUT: congenital anomalies of the kidney and urinary tract; LLAC: lupus like anticoagulant.

CKD is a risk factor for adverse pregnancy outcomes in all its stages, meaning that the risk is higher than in a pregnancy occurring in a woman without CKD even without hypertension and relevant proteinuria and in the presence of a normal kidney function, as it happens, for instance, in interstitial diseases, glomerulonephritis in remission or early-stage polycystic kidney disease [44, 49, 52–58].

The ‘quantity’ of kidney tissue is a relevant issue since a significant increase in the risk of preeclampsia (mainly late) and of preterm delivery is described in kidney donors and women with congenital solitary kidney [59–61]. It is interesting to remark that in kidney donors the risk of preeclampsia, the main ‘marker’ of adverse pregnancy outcomes in the overall population, is increased with respect to the pre-donation phase, but is still in the range of the overall population, further stressing the importance of ‘global’ health (as required for becoming a kidney donor) in determining pregnancy outcomes [59–61].

The need to prepare for pregnancy, pursuing the best possible control of the kidney disease (e.g. immunologic balance) and of its complications (e.g. high blood pressure), is obvious but, as previously discussed, often unmet.

## MAIN KIDNEY-RELATED AND UNRELATED RISK MODULATORS FOR PREGNANCY

The single parameter that is probably the most relevant in pregnancy is kidney function. Even minor differences in kidney function (for example, stage 2 versus stage 1) increase the risk of adverse pregnancy-related outcomes [63]. Risk increases along with kidney function impairment [63]. Once pregnant, at least in patients with reduced kidney function or kidney transplantation decrease in serum creatinine, a preserved hyperfiltration response is associated with better pregnancy outcomes.

Baseline hypertension and baseline proteinuria are also associated with an increased risk of adverse pregnancy outcomes

[6, 44, 54, 55, 62, 64]. The extent and the mutual interaction between them and with kidney function still need to be established. A schematic attempt to empirically stratify in three ‘large’ risk groups (low, medium and high) is reported in Table 4.

**Table 4:** An attempt to graduate risk factors for adverse pregnancy outcomes in a woman with CKD.

Risk factor	Mildly increased risk	Moderately increased risk	Highly increased risk
Blood pressure	Normal	Drug controlled	Poorly controlled
Proteinuria	None	>0.5 g/day	>1 g/day
Kidney function	eGFR >90 mL/min/1.73 m^2^	eGFR <90–60 mL/min/1.73 m^2^	eGFR <60 mL/min/1.73 m^2^
Diabetes	No	Well controlled	Poorly controlled
Autoimmune disease	No	In remission	Active and/or flares and/or occurring during pregnancy
Obesity	No	Moderate	Severe
Type of disease (examples)	Interstitial diseases/kidney scars	Immunologic diseases in remission, kidney transplant in stable conditions	Active immunologic diseases; kidney transplant with reduced kidney function

Modified from KDIGO 2021 Clinical Practice Guideline for the Management of Glomerular Diseases.

eGFR: estimated glomerular filtration rate.

The main non-renal risk modifiers are reported in Table 5 [65–79].

**Table 5:** Main ‘non renal’ risk modulators in pregnancy.

Risk modulators	Comments
Age	Teenagers or women over 40 years old are at higher risk of pregnancy complications [65, 66]. Teenage pregnancies are associated with belonging to a socially deprived milieu
Ethnicity	Black, Hispanic and overall minorities have a higher risk or adverse pregnancy-related outcomes; whether this is a reflection of socioeconomic conditions is controversial [67–69]
Low birth weight	The risk is reported to be increased staring from a birth weight <2500 g, but is probably a continuum [70]
Previous complicated pregnancies	Probably a continuum, in dependence from the number and severity of previous pregnancy complications [71]
Social status	Higher risk is associated with lower social status [72]
Immunologic diseases	All immunologic diseases, even in the absence of kidney disease, are associated with a higher risk of adverse pregnancy outcomes [73]
Baseline hypertension and diabetes	The risk is increased also in the absence of overt kidney disease or end-organ damage, and is increased in poorly controlled conditions [74]
Obesity and overweight	A continuum of increased risk is associated with overweight and obesity. Weight loss before pregnancy and low weight gain in pregnancy may improve outcomes [75, 76]
Anorexia and eating disorders	Less extensively studied than obesity, but the risks are likely to be increased [77]
Alcohol and drug abuse	The fetal alcohol syndrome is well-known. Toxicity and deprivation are often combined in illicit street drug abuse [78]
Assisted fertilization	Risk increases with the complexity of the procedure and is the highest in egg donation [79]

Many of these risks are non-modifiable (age, ethnicity, low birth weight). Their acknowledgement is, however, essential for establishing a follow-up plan in pregnancy, and may be relevant in the discussion about the risks of adverse pregnancy outcomes.

## MAIN RISKS FOR KIDNEY FUNCTION REDUCTION

In general, it is held that pregnancy in a woman with CKD stage 1, normal blood pressure and no relevant proteinuria at baseline (i.e. also at low risk of adverse pregnancy outcomes) is not accompanied by a risk of worsening of the kidney function, but that worsening of the kidney function is common in women who start pregnancy with severely impaired function, or high-level proteinuria [44, 54, 55, 80–92]. Hence, at least in the advanced CKD stages, counselling should include a discussion of the need for starting dialysis, and of providing a vascular access.

More recently, some data, mainly from immunoglobulin A (IgA) nephropathy, suggest that a pregnancy in which a hypertensive disorder of pregnancy or preeclampsia occurs is a risk factor for worsening kidney function even in previously stable patients [48, 93–95]. Large-scale data are not available, and quantifying the risk is impossible; intensifying follow-up of patients with CKD after a hypertensive disorder of pregnancy is an option that should be discussed with the patient.

## MAIN RISKS FOR THE CHILD

Prematurity, defined as birth before completing 37 gestational weeks, is relatively frequent, but late-preterm delivery is, at least in highly resourced settings, associated with an only mild increase in both short- and long-term risk (Table 3). However, early preterm, now often defined as delivery of a live-born baby before 32 completed gestational weeks, acknowledging the improvement in prenatal care in preterm babies, and extremely (or very early) preterm birth (defined as before 28 completed gestational weeks) is still associated with relevant morbidity in both the short term (major threats being cerebral haemorrhage and sepsis) and long term (metabolic diseases and CKD) [96]. The risk of prematurity increases with the worsening of kidney function.

There is no clear threshold of birth weight below which risks both in the short and long-term increase, and the cut-points at 1500 and 2500 g are usually retained (Table 3). Very early preterm delivery and very low birth weight are also associated with neonatal death. The risk of prenatal death and stillbirth inherent in diabetes and immunologic diseases, albeit not specifically increased by the presence of CKD, also need to be explained to patients.

Glomerular formation and maturation occur in the late phases of gestation, and this explains the association between prematurity and reduced nephron count, often leading to an increase in the incidence of hypertension and CKD in adulthood [97–101]. Likewise, females born very premature or with a low birth way may have a higher risk of developing preeclampsia in their pregnancies, thus perpetuating the vicious cycle of small babies, leading to small babies [97]. The late maturation of adipose tissue is the basis of an increased risk of obesity and metabolic diseases, which can further challenge kidney function [97]. From the cognitive point of view, although limited studies on the topic are available, the evidence suggests that having a mother with CKD does not inevitably compromise a child's chances of attaining the usual developmental milestones and, once more, the risks, only partially known, are mainly linked to prematurity and possibly enhanced by intrauterine growth restriction.

## COUNSELLING TO GUIDE INTERVENTIONS

Mitigating pregnancy-related risks in women with CKD requires a nuanced and comprehensive approach encompassing pharmacologic interventions and dietary strategies. Optimization of blood pressure control is cited by all guidelines/best practices, even if the target is not fully agreed on; levels below 130/80 mmHg are probably sound [7–10].

Discontinuation of potentially teratogen drugs is obviously recommended [7–10]. Supplementary data, Table S1 summarizes data on frequently used drugs in women with CKD [7, 102]. Some issues remain open, including delaying the discontinuation of some potentially teratogen drugs, including angiotensin-converting enzyme inhibitors and mycophenolate (Supplementary data, Table S1).

Low-dose aspirin, from 75 to 150 mg, is now routinely employed to prevent preeclampsia in high-risk pregnancies. The indications for when the therapy should be started varies, ranging from a positive pregnancy test to 12 weeks of gestation, and discontinuation is advised from 28 weeks until the end of gestation; local protocols and logistic aspects should guide these decisions [7–10]. In settings with timely access to ultrasound confirmation and dating of pregnancy, waiting for pregnancy confirmation before starting low-dose aspirin is the preferred policy, while starting at a positive pregnancy test may be wiser where access is limited or late. Since, at least in some countries, aspirin boxes carry a warning against use in pregnancy, patients should be advised that the warning should not frighten them or discourage use for prevention of preeclampsia.

Dietary interventions should aim to a balanced and kidney-friendly diet. A moderate limitation of protein intake and a plant-based diet can mitigate the hyperfiltration observed during pregnancy, and can be used safely in pregnant women with proteinuria or advanced CKD [104–106].

Obviously, all the strategies advised in ‘healthy’ pregnancies, including folic acid during preconception, and correction of vitamin D or low calcium intakes should also be undertaken in CKD patients [103].

Pregnancy may be very time-demanding in a woman with CKD. Frequent monitoring plays a crucial role in CKD pregnancies, and should be mentioned at preconception counselling. The Italian best practice sets indications on the minimum frequency of clinical controls (monthly) and all available guidelines underline the importance of controls and of the setting of care. The frequency has to be further modulated by the presence of hypertension, proteinuria and by CKD stage, together with the concomitant comorbidity profile [7–10, 12, 13]. Likewise, the possibility of needing frequent controls of fetal development should be underlined, as should the importance of the differential diagnosis between preeclampsia and CKD in case of hypertension and proteinuria, supported, whenever possible, by the monitoring of angiogenic–anti-angiogenic placental biomarkers [107]. In late CKD stages, the discussion should also focus on the possibility to need to start dialysis, on dialysis modalities and schedules, and on the preferred vascular access.

Once more, the balance between stressing an already anxious patient, asking her to undergo frequent controls, and the importance of underlining the need for strictly monitoring pregnancy is not always easy to achieve and is probably impossible to attain in all patients. However, reassuring our patients that even though we cannot guarantee results, we can generally deliver excellent care and personalize follow-up can be a part of a non-dogmatic and flexible counselling approach.

## SETTING PRE-PREGNANCY GOALS

There is no ‘recipe’ or fixed sequence of questions that best manage pre-conception counselling. How the colloquium is run depends on several factors, including the patient's priorities, questions, clinical and psychological situation, age, and comorbidity.

In Table 6, we have summarized the main issues and a hypothetical sequence of topics and questions that, based upon our experience, can serve as a checklist for counselling.

**Table 6:** Steps suggested in pre-pregnancy counselling in women living with CKD.

Issue	Comments
Ask the patient about her priorities and doubts, and about what she knows (or thinks she knows) about her kidney disease and about pregnancy	This first phase is a moment of mutual exchange and underlines the need for a personal approach to counselling. Counselling does not concern a disease; counselling regards an individual person, with a history (not just of kidney disease), a culture, experiences and projects
Discuss reproductive freedom	Especially important if the woman seems concerned about her ‘right’ to become a mother, or if the interviewer feels that motherhood is seen as a social need more than as a personal aspiration
Start from ‘the bright side’, considering that many women have been warned against pregnancy before coming to speak with you, and discuss the possibility of becoming pregnant and having a successful pregnancy	Highlight that pregnancy can be successful in all CKD stages, including on dialysis but that success is higher in ‘well prepared’ cases, especially in advanced CKD. Highlight the need for personal commitment to repeated testing, clinical controls dietary management and therapies, but that these are all aimed at improving pregnancy outcomes, and that this is a shared journey
Discuss risks extensively	Focus not only on what is known, but also on the uncertainties, due to the heterogeneity of CKD
Plan to evaluate the clinical situation in detail and to minimize risks	This includes, but is not limited to, imaging, blood pressure control, other tests according to the underlying disease and optimization of blood pressure control, nutritional status and diabetes, if present, and drug management
Define the best timing together with the patient, setting reasonable goals	The definition of reasonable goals is important: it has to be contextualized to each case
Plan a strategy of controls during pregnancy	Even in the early CKD stages, some experts suggest at least monthly controls; the frequency of controls increases along with the severity of the kidney disease and in the presence of hypertension or proteinuria. The possibility of dialysis start in advanced CKD and the choice of vascular access should also be discussed
Explain that some drugs and supplements are, or may be needed in pregnancy, and others have to be discontinued	Drug management should be reviewed in pregnancy and the timing for discontinuation of some drugs is still a matter of discussion. Beside the optimization of present treatments, discuss that need for pregnancy-related ones (aspirin, vitamins), or on the way to face pregnancy-related problems (hyperemesis, hypertensions)
Refer for evaluation of fertility if appropriate	For example age near the ‘limit’ of 40 years; previous treatment with cyclophosphamide or other toxic drugs; a history of at least 6 months of unprotected intercourse, advanced age or clinical problems of the partner may lead to consider a timely evaluation and, if needed, care

Patients need to be involved for being able of setting personalized goals [35].

For example, obesity is a risk factor for pregnancy and this risk is added to the risk inherent to CKD [76]. However, for an obese woman losing weight is a difficult task, and setting too low a weight level to reach before pregnancy may prove to be frustrating and useless. Conversely, small advances, and positive reinforcement, may favour empowerment and help weight control during pregnancy, which is probably even more crucial for pregnancy outcomes [108].

Conversely, for example in diabetes since malformations in type 1 diabetic women also increase in relation to diabetes control, and since attaining good diabetes control (for example changing insulin schedules) is a goal attainable in a few months in most cases, focusing on this attainable goal should be a priority [46, 47].

The age issue is also important: in a CKD woman in the 35–40 years age group, fertility is likely to drop rapidly, and this may need to be balanced, for example in the decision to undertake a pregnancy with advanced CKD or after kidney transplantation (especially in the absence of a living kidney donor) [109].

## SCENARIO-BASED COUNSELLING

**Figure 4:**
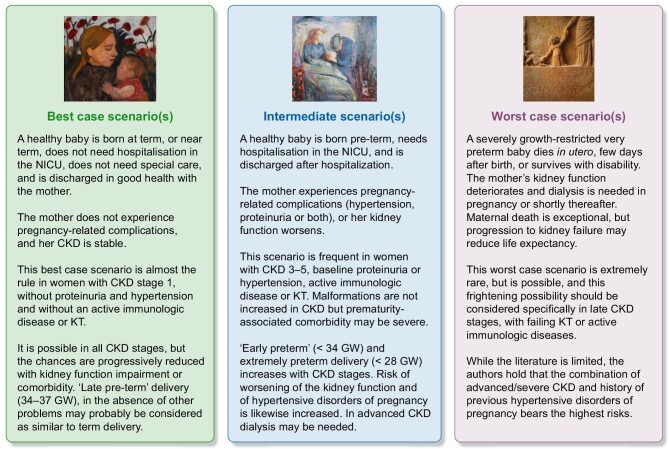
Counselling on a ‘case-based scenario’.

In such a context in which we have to convey not only knowledge, but also uncertainties, a scenario-based approach (Fig. 4) may be useful.

This type of approach does not rely on fixed numbers, but first describes the ‘best-case’ scenario, which is the one that is most frequently encountered by far, at least in settings in which all CKD patients, including those with milder forms of the disease are followed up.

The intermediate-case and the worst-case scenario are further matters for discussion, and the emphasis on the intermediate case scenario should probably be modulated by the patient's kidney situation and extra-renal comorbidity. Mentioning the worst case scenario can be difficult, even if many women who are referred for a specialist consultation have already been faced by discouraging opinions, and discussing also the worse possibilities may have a positive effect. We often highlight that the maternal risks of death are not even assessable in women on follow-up and without uncontrolled diseases at start of pregnancy, and summarize the risks for the baby in one question: ‘would you be able to face it?’ Examples are often received better than numbers, and may increase understanding of the risk of failure, without entailing losing the hope of success.

## POST-PREGNANCY COUNSELLING

Pregnancy is a stress test for the mother, and the results of pregnancy may be seen as a window on her future health, as well as for the health of her baby [110, 111].

Hence, it may be important to consider, besides pre-pregnancy counselling, the option of post-pregnancy counselling, discussing how pregnancy outcomes can be expected to modulate future maternal or child's health.

Starting from the positive side, the best-case scenario is associated with no increased long-term health risk for the mother, as compared with disease-matched peers, and for the baby. This is also a situation in which a further pregnancy, if the situation remains stable and age limits are not reached, has probably the highest odds of repeat success.

In the intermediate-case scenario, in which complications occurs, their effects should be graduated according to their type and severity. We have some data on recurrence of preeclampsia and hypertensive disorders of pregnancy, even in the context of very discordant estimates (from 15% to over 70%) but no study is focused on recurrent complications in CKD pregnancies [71, 112]. Limited evidence in IgA nephropathy, however, suggests that patients with CKD may experience an accelerated course if they have a complicated pregnancy; similar data were, however, not recorded after kidney transplantation in a large Australian cohort [93, 113]. However, even in the absence of sound evidence, intensifying follow-up after a complicated pregnancy may be a reasonable strategy for improving long-term outcomes.

Discussing the risks children may encounter is even more delicate. Once more, this requires time and tact, but since acknowledging risks may improve outcomes, this difficult task should become a part of the discussion with parents, before and after pregnancy.

For children born small, and/or preterm, controlling weight and avoiding obesity is a major and attainable goal, and warning the mother is usually simple; avoiding overweight may also be a further argument to support breastfeeding. A highly sensitive issue regards information about the risk of a ‘small’ female baby to develop preeclampsia once pregnant. Timing of the information is important and depends also upon planned maternal follow-up, and can be postponed in women strictly followed. In the experience of the authors of this review, due to the frequent loss to follow-up of women with mild CKD or evaluated after a hypertensive disorder of pregnancy, we tend to deliver the information early, in the context of a discussion on the implications of pregnancy outcome for maternal health. Underlining the positive aspects is important: since preeclampsia may be at least in part prevented by appropriate care, or better managed if detected early, a possible approach is telling mothers that their ‘small’ daughters will require attention in pregnancy, to allow prevention of pregnancy complications, a goal that will surely improve over time [70, 97].

## CONCLUSIONS

Women with CKD can have a successful pregnancy in any CKD stages and, as a general rule, pregnancy should not be contraindicated in CKD.

While the general concepts of an increased risk in patients with severe CKD, high-level proteinuria and hypertension are well established, the entity of the risks also depends upon the type of disease, patient adherence, setting and availability of care. Due to the high heterogeneity of kidney diseases, stages, grades and settings of care, giving precise figures is usually impossible, and a discussion of the various possible scenarios may be more informative and helpful to improve coping strategies. Furthermore, since pregnancy is a window on future health, pre-pregnancy counselling should be integrated by a post-pregnancy counselling aimed to optimizing care for the mother and the baby after delivery.

## Supplementary Material

sfae084_Supplemental_File

## Data Availability

No new data were generated or analysed in support of this research.
