# Supplementary material for: Pre-gestational counselling for women living with CKD: starting from the bright side
Source: Clin Kidney J. 2024 Mar 22;17(5):sfae084. doi: 10.1093/ckj/sfae084 (PMC11070880; doi:10.1093/ckj/sfae084)
Supplement: sfae084_Supplemental_File [file sfae084_supplemental_file.docx]

Supplementary Table 1:

Safety in pregnancy of some commonly used drugs for women with CKD

| **Drug** | **Risk in pregnancy** | **Suggestions** | **Safety according to FDA** |
| --- | --- | --- | --- |
| **Immunosuppressive drugs** | | | |
| Azathioprine | Occasional occurrences of temporary leukopenia and/or thrombo-cytopenia are uncommon when maternal white blood cell counts are maintained within the normal range. | The azathioprine dosage should be adjusted, keeping maternal white blood cell counts in range. | D |
| Belimumab | Insufficient evidence. | Avoid if possible. | C |
| Calcineurin inhibitors (Cyclosporine-Tacrolimus) | The risks reported (low birth weight, preterm delivery and IUGR) could be disease related. | Dosing serum unbound drug- | C |
| Cyclophosphamide | Miscarriages and malformations are reported. | Discontinue in first trimester; conditional use in second and third trimesters when risks are lower | D |
| Eculizumab | Insufficient evidence | Avoid if possible | C |
| Hydroxychloroquine | Safe in pregnancy | Continue or start | B |
| Intravenous Immunoglobulins | Insufficient evidence | Avoid if possible | C |
| Mycophenolate Mofetil | Malformations and pregnancy loss | Discontinue (from 6 weeks to 6 months before conception) | D |
| Methotrexate | Mutagenicity and teratogenicity reported | Discontinue (timing for discontinuation is not clear) | X |
| M-Tor inhibitors (Sirolimus-Everolimus) | Insufficient evidence, but animal models suggest preterm birth, fetal mortality and reduced fetal weight | Discontinue 12 weeks before conception for Sirolimus and 8 weeks for Everolimus | C |
| Rituximab | Lower white cell count in neonates (generally normalized within 6 months) | Conditionally continue | C |
| Steroids | The risks (IUGR, low birth weight, transitory immune depression in the neonate, miscarriages, intrauterine death) seem to be dose or disease related  Contradictory data regarding cleft lip and cleft palate. | Limit the dosage and duration of exposure. | C |
| **Antihypertensive drugs** | | | |
| ACE-I and ARB | Risk of malformations | Discontinue | C (1^st^ trimester)  D (2^nd^-3^rd^ trimester) |
| Alpha blockers | Insufficient evidence | Second choice | C |
| Alpha-methyl dopa | Negative effects are not reported | First choice | B |
| Beta blockers:  Atenolole  Pindolole  Metoprolol | Older studies report fetal growth restriction; they may induce mild and transient hypotension, bradycardia and hypoglycaemia at delivery | Second choice | D  B  C |
| Clonidine | Slowing fetal growth is occasionally reported | Second choice | C |
| Labetalole | Negative effects are not reported | First choice | C |
| Niphedipine | Negative effects are not reported | First choice | C |
| Short acting niphedipine | Risk of severe hypotension with negative effects on placental flows | Discontinue | D |
| **Other drugs** | | | |
| Acetylsalicylate | Negative effects are not reported | Discontinue before delivery | NC |
| Erythropoietin | Does not cross the human placenta | Continue | C |
| Low molecular heparin | Does not cross the human placenta; may increase bleeding risk at the utero-placental junction | Continue in prophylaxis and treatment of thromboembolic complications during pregnancy. | C |

Legend: FDA : Food and Drug Administration ; FDA rating : B, no evidence of risk in humans ; C, risk cannot be ruled out ; D, positive evidence of risk ; X, contraindicated in pregnancy. NC, not classified
